# Experience Them, Love Them, Protect Them—Has the COVID-19 Pandemic Changed People’s Perception of Urban and Suburban Green Spaces and Their Conservation Targets?

**DOI:** 10.1007/s00267-022-01721-9

**Published:** 2022-10-12

**Authors:** Donna Tansil, Christian Plecak, Karolina Taczanowska, Alexandra Jiricka-Pürrer

**Affiliations:** 1 Department of Landscape, Spatial and Infrastructure Sciences, Institute of Landscape Development, Recreation and Conservation Planning, University of Natural Resources and Life Sciences Vienna, Vienna, Austria; 2 Department of Botany and Biodiversity Research, University of Vienna, Vienna, Austria

**Keywords:** Urban green areas, Protected areas, Urban forests, COVID-19, Environmental perception, Austria

## Abstract

Public green and open spaces fulfil various social, ecological, economic, and aesthetic roles, which can be complementary while also competing with one another. The COVID-19 pandemic catalysed multiple societal changes, including citizens’ perception, needs and expectations relating to urban green spaces. This article discusses the extent to which the temporally and geographically changed patterns of experiencing these natural spaces also influenced users’ perception and behaviour as well as their appreciation of the conservation areas. The study is based upon two surveys carried out in the greater metropolitan region of Vienna, the capital city of Austria. A quantitative survey (representative online panel) among Viennese population (*n* = 1012), as well as qualitive interviews with experts responsible for conservation areas, administrators of federal parks, along with NGOs representatives were carried out in spring and summer 2021. Our study shows changed perception of urban citizens towards green spaces during the COVID-19 pandemic. An increased importance of time spent in nature (68%) and possibility to visit large green areas (67%) was reported by Viennese citizens. Also, higher recognition of green spaces located close to home was observed among 69% of the respondents. There were significant differences in opinions on green areas during the pandemic in various age and gender groups. Thus, the presented study contributes to the ongoing international discussion on the transition of societal needs and its effects on urban green spaces induced by the pandemic. Presented results highlight the need of urgent transformation towards a more sustainable, resilient and healthy urban space.

**Figure:**
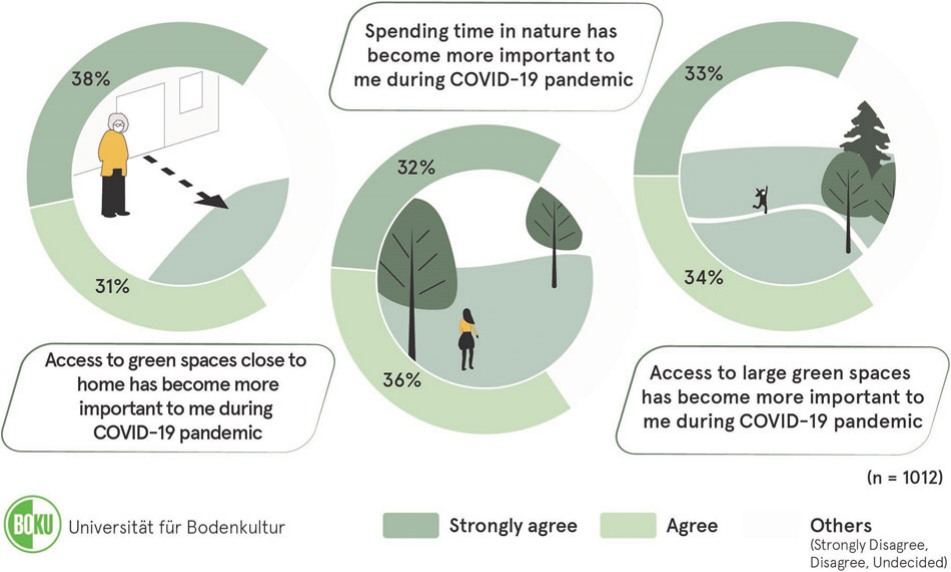
Graphical abstract

Graphical abstract

## Introduction

The positive effects of public green and open spaces are undisputed, particularly in urban and suburban areas. They fulfil various social, ecological, economic, and aesthetic roles, which complement each other, but can also compete with one another. Given the complexity of our individual needs and requirements (Tessin 2011), a great deal is demanded of public green and open spaces in urban areas. Green and open spaces networks are designed to protect or enhance natural resources while at the same time to provide space for recreational use, social interaction and active mobility (Thompson 2002). These requirements have been altered by the COVID-19 pandemic and the various measures implemented in Europe since the beginning of 2020 to contain it. New patterns developed in the use of green spaces as a result of lifestyle changes, such as (increased) remote work, home-schooling, and caring for nursery-aged children at home, as well as limits to people’s personal range of movement due to lockdowns, self-isolation rules, and closed leisure facilities (Astell-Burt and Feng 2021; Ugolini et al. 2020). During the pandemic, urban and suburban green spaces fulfilled a variety of needs. Studies, such as the one conducted by Robinson et al. (2020), confirm that people visited green spaces in order to improve their personal well-being. In a study carried out by Atalan (2020), the majority of participants indicated that nature had helped them cope with the pandemic. Soga et al. (2021) investigated the extent to which personal contentment and subjective well-being were connected to people’s distance to green and blue spaces as well as their access to them during the pandemic. Partly this alternated interest in green areas and nature overall influenced also the visiting rates and behaviour in conservation areas.

Conservation areas experienced highly heterogeneous developments across the world. Previously published studies have demonstrated a multifaceted picture concerning the impact of the global pandemic on conservation areas as well as the pursuit of conservation objectives. On the one hand, the decline in economic activity as well as private transport generally reduced the pressure placed on natural spaces by contaminants, noise pollution, and other sources of disruption, but on the other hand, financial revenues generated by tourism in conservation areas decreased at the same time. This had a somewhat negative impact, as fewer resources were available for poaching checks (Buckley 2020). The studies also revealed unexpected effects, i.e., on noise levels during lockdown periods. As such, for example, significantly higher decibel levels could be measured at the periphery of conservation areas in the United States where there are passing expressways, which can be attributed to the fact that traffic restrictions enabled the remaining drivers to drive more quickly (Terry et al. 2021). In contrast, in other remote and suburban natural spaces, lower volume levels were detected as a result of reduced mobility (Terry et al. 2021). These factors had a somewhat significant impact on the communication, interaction, behaviour, and fitness of many wild animal populations of different species. This effect’s degree of impact differed according to the prevalent season during the lockdowns and the associated vegetation foliage (Terry et al. 2021). Wild animals created new, temporary habitats close to conservation areas (Corlett et al. 2020). These developments were particularly prevalent in the first phases of the pandemic as well as during periods with strict lockdown measures.

While negative consequences like poaching were increasingly brought about by the significant decrease in tourism, or complete absence thereof, in some areas (Buckley 2020), other places were strongly impacted by heightened amounts of litter (Bates et al. 2020) as well as the changed number of visitors. By contrast, it was possible to observe greater usage and influence of conservation areas throughout the course of the pandemic, particularly in urban and suburban areas. Studies, such as the one conducted by Ugolini et al. 2020, show that the opportunity to avoid large gatherings of people in order to minimise the risk of infection brought the importance of large green spaces and expansive areas to the fore (Ugolini et al. 2020). As a consequence, initial studies recorded several detrimental effects in suburban conservation areas. One common effect caused by the use of urban conservation areas during lockdown periods was the development of new paths which drove the fragmentation of wildlife habitats and sometimes led to the creation of 30% more paths. Consequently, vegetation and wild animals were partly driven away. Both hikers and cyclists created new paths so that they could avoid excessive numbers of visitors (Primack and Terry 2021). Taking earlier studies on these topics into consideration, dog owners who did not keep their dogs on a leash, as is mandatory, also contributed to considerable damage to individual populations, especially during breeding seasons (Miller et al. 2001; Reed and Merenlender 2011; Bötsch et al. 2018). Moreover, changes were also recorded to the microclimate in the field of erosion control. Additionally, invasive species were provided with new habitat opportunities as a result (Primack and Terry 2021).

Even prior to the pandemic, there were conflicts over usage due to the diverse demands placed on green spaces, activities, and visitors’ needs (Schneider 2000, Arnberger et al. 2002, Vaske et al. 2007). Conservation areas have embraced these challenges by means of diverse approaches to visitor management. For them, early conflict prevention as well as effective visitor management are of particular importance due to their objectives concerning species protection and promotion of biodiversity. International studies show the increased importance of adaptive visitor management in suburban conservation areas in containing the spread of COVID-19 and highlight its central role in enabling the continued pursuit of conservation targets to the greatest extent possible (Ma et al. 2021).

Prior to the pandemic, numerous international studies investigated the degree to which green spaces are evaluated according to functional significance and other aspects, which are conductive to individual well-being on the one hand and influence social targets such as maintaining biodiversity on the other. Studies showed that aesthetic appreciation and environmental awareness play a more important role in conservation areas in the suburbs than in the more highly populated urban areas, where green spaces are more commonly utilised to strengthen social relationships and cultural diversity (Riechers et al. 2019). Furthermore, the studies discovered that also home gardens fulfilled multiple cultural ecosystem services, such as aesthetic and spiritual experiences, outdoor recreation and hobby, space of social relations, contact with nature, maintenance of traditional ecological knowledge and environmental education (Calvet-Mir et al. 2012).

In research carried out over several decades concerning the communication of conservation objectives and environmental education, it was found that both autonomous contact to nature as well as independently experiencing and discovering natural spaces are two of environmental education’s central elements (Hart and Nolan 1999; Hutchinson 1997). While school and educational projects had to be put on hold or reduced in conservation areas due to the pandemic, along with research projects and monitoring programmes (Smith et al. 2021), people’s experience of nature intensified due to the absence of other leisure activities and their changed everyday life. This was particularly true for families with children and the younger generations. Initial studies show that green spaces, including conservation areas in the suburbs, experienced greater use because of the pandemic (Astell-Burt and Feng 2021; Ugolini et al. 2020).

This article discusses the extent to which the temporally and geographically changed patterns of experiencing these natural spaces also influenced users’ perception and behaviour as well as their appreciation of the conservation areas. It presents a summary of two surveys carried out in the greater metropolitan region of Vienna. A representative quantitative survey of the metropolitan region of Vienna with its population of two million as well as a qualitive investigation into this area by means of structured interviews with those responsible for conservation areas, administrators of large green spaces (federal parks), and NGOs provided the data basis.

As a result, this article investigates: (i) the extent to which the management of conservation areas in and close to a large metropolitan region observed pandemic-related impacts on usage behaviour as well as the perception of nature and conservation targets, and (ii) the altered relevance of green spaces close to home, particularly large green spaces, for those living in this capital region.

## Methods

### Case Study Area

The presented findings are based upon a case study carried out in the City of Vienna (the capital city of Austria) and the surrounding municipalities. It focuses on a variety of green and open spaces, including protected areas.

In our study region, there are five large natural landscapes that are classified as Natura 2000, national parks, biosphere reserves and/or wildlife parks (Bisamberg, Marchfeld, the Vienna Woods, the Danube area, terraced landscapes to the south). Although most large conservation areas are typically located in the outskirts of the city, some do exist in an immediate proximity of densely populated urban areas (e.g. Vienna Woods/Wienerwald Biosphere Reserve).

**Fig. 1:**
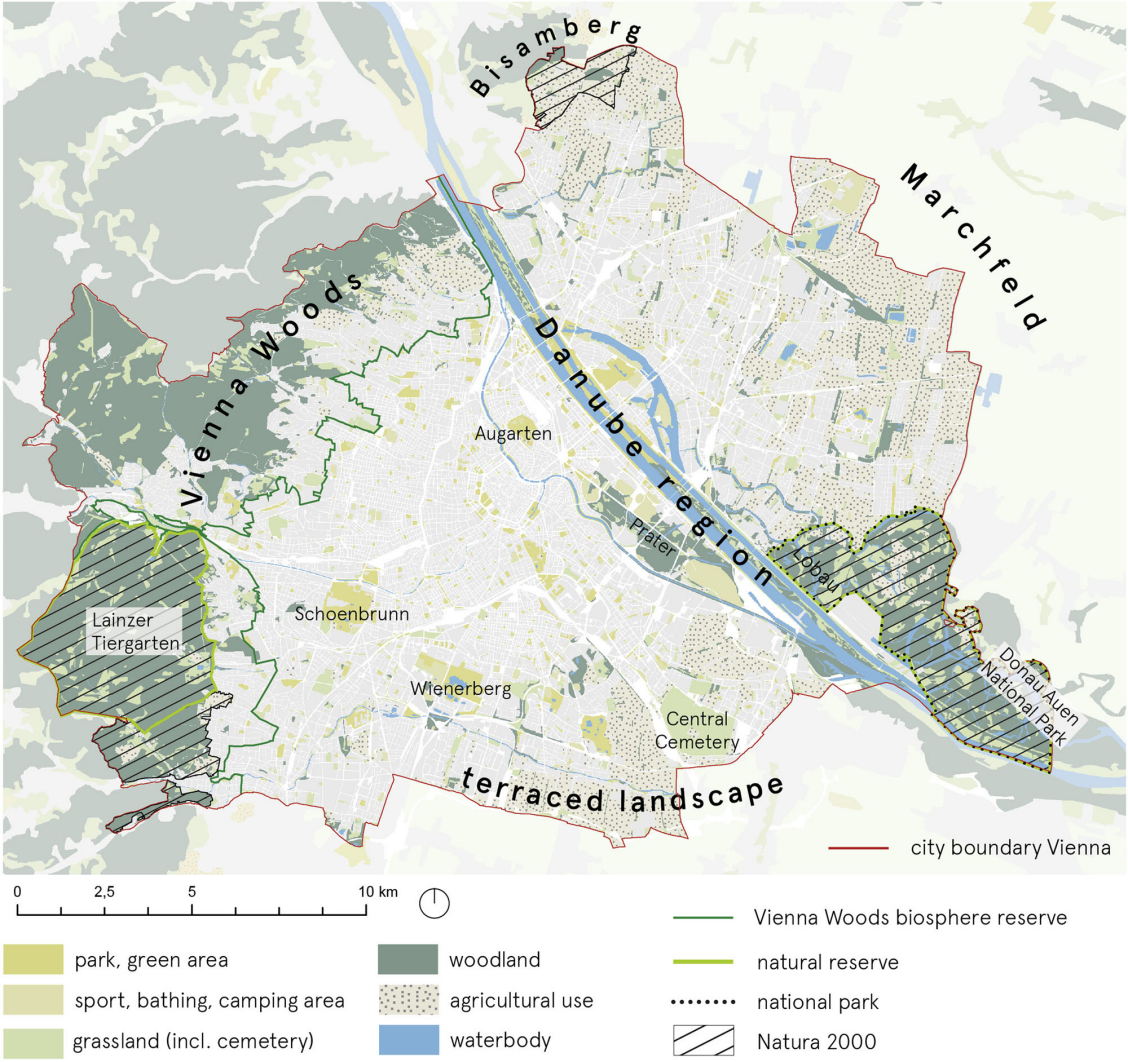
The location of major green areas in the City of Vienna [Geodata credit: basemap.at; Corine Land Cover (CLC) 2018 version 2020_20u1—European Environment Agency (EEA) under the framework of the Copernicus programme—CLC dataset produced with funding by the European Union; city boundary, Viennese protected areas boundaries -Stadt Wien – https://data.wien.gv.at; map created using ESRI ArcGIS]

In terms of non-conservation open space, there is a diversity of these across Vienna’s districts. Together with conservation land, large parks shape the city’s green character. At more than 50%, the proportion of green spaces in the City of Vienna makes it one of Europe’s greenest cities (STEP 2025). Many of these large green spaces, such as the Lainzer Tiergarten, Lobau, Prater, Schoenbrunn and Augarten, can be attributed to the days of the Habsburg Monarchy. Moreover, Red Vienna’s housing projects and the closure of cemeteries secured further green spaces for the city’s inhabitants (Wieshofer et al. 2015). Unfortunately, these green spaces are not evenly distributed throughout the City of Vienna. As an instrument for the future development and maintenance of Vienna’s green and open spaces, the functional concept for green and open spaces, which is part of the City of Vienna’s urban development plan (STEP 2025), states key figures for the provision of green and open spaces related to their quantity, quality along with spatial connectivity. Figure 1 illustrates the location of major green areas in the study area.

### Data Collection

Both quantitative and qualitative survey methods were used in this study. Quantitative survey (online panel survey) was selected to examine the views of inhabitants who use conservation areas and other urban green spaces, while a qualitative approach (structured expert interviews) was used to investigate the perspective of authorities responsible for maintenance and management of those sites.

Data collection was carried out in the early summer of 2021. At this time, many public health measures were still in place in Austria to contain the second and third waves of the COVID-19 pandemic. The previous full lockdown in Vienna and the surrounding area was around one month earlier. Food service outlets and leisure facilities had only recently been opened to the public subject to certain conditions (proof of a negative test, recovery, or vaccination).

#### Quantitative Survey

Online survey technique was applied to investigate Viennese population perspective. Lime Survey was used to design survey form and corresponding database. The respondents were recruited via commercial online panel Marketagent. 1012 interviewees completed entire questionnaire. Partially completed (but eventually non-submitted surveys) were not considered in the analysis. The responses were anonymous (no personal data nor IP-addresses of the respondents were stored in Lime Survey database).

In order to obtain a representative sample reflective of the population’s characteristics, a (stratified) random sample was selected by applying the quota method. Pre-defined quotas for gender, age and regional distribution of the population were considered during data collection within online survey panel. Target sample included 51% female and 49% male respondents. Participation was open to the respondents aged 16 years or more. 2.5% of the sample were in the age bracket 16–18, 38.3% were aged 19–39, 39.7% were between 40 and 64 years of age, and 19.5% of participants were over 65. The quotas were also aligned with the population distribution in the Viennese districts and the surrounding communities. Seven regional clusters were considered in the target sample design: C1 Vienna Centre (15.5%); C2 Vienna Central-East (9.4%); C3 Vienna West (24.2%); C4 Vienna South (24.8%); C5 Vienna North-East (17.2%); C6 Surrounding communities North-East (3.5%); C7 Surrounding communities South-West (5.4%).

The online standardized questionnaire captured socio-demographic characteristics of the respondents (gender, age, place of living, education and occupation status) as well as behavioural and psychographic data related to green spaces. List of survey questions related to the presented results can be found supplementary material (S1).

#### Qualitative Interviews

A total of ten structured interviews were carried out with experts from the management of conservation areas, two NGOs which host numerous events on environmental education and communication, including in conservation areas, as well as the administrative department responsible for Vienna’s green spaces. The conservation areas whose managers were interviewed were of various conservation status. They included a biosphere reserve, a Natura 2000 site, a landscape conservation area, and a wildlife park.

The structured interviews comprised three main topics:

- Changes and their effects: changed flow of visitors (frequency, distribution, seasonal distribution, etc.) and implications for the conservation areas and large green spaces
- Adapted perception of conservation areas and large green spaces in urban/suburban areas, conservation targets, and the regions’ cultural ecosystem services.
- Future perspectives: lessons learned in terms of visitor management, future environmental communication offers, potential future interest in conservation, and activities in this field.

### Data Analysis

Quantitative data were analysed using statistical procedures in R. Likert Package was used to analyse and illustrate the descriptive results. Additionally, statistical tests: Chi-Square Test (in case of cross-table frequencies >5) or Fisher Exact Test (in case of cross-table frequencies were equal or less than 5 were used to determine, if there were significant differences in opinions among specified respondent groups. Statistical tests considered all valid opinions of the respondents. The answer category “I don’t know/I have no opinion” was not considered neither in Chi-Square Test nor Fisher Exact Test, therefore in some analysis sample size varies from the entire number of completed surveys (*n* = 1012). Number of valid cases considered in specific analysis is reported in the results section (figure descriptions).

Spatial data (reported point locations of visited green areas) were analysed in ArcGIS software using Kernel density algorithm. The following parameters were used in the calculation: grid resolution: 50 meters; search radius: 400 meters; area units: square meters.

Qualitative interviews were transcribed, coded according to major categories (see listing in Table 1) and sub-codes. The major categories reflected the core questions’ thematic orientation and sub-codes were identified by analysis of the entire interview transcripts.

**Table 1:** Overview of major categories and sub-codes used in analysis of qualitative interviews

*Categories*
A_changed visitor numbers and behaviour
B_Impacts on the protected area overall
C_Impacts on conservation targets
D_Challenges for visitor management
E_Development of new strategies for visitor management
F_Changed perception of the ecosystem services of the protected area
G_Changes in interest in nature conservation (targets)
H_Novel strategies/ adaptation of strategies in protected area management

Sub codes comprised for category A (changed visitor numbers and behaviour) for instance A_1 frequency, A_2 spatial distribution, A_3 seasonal distribution, A_4 changes in distribution between weekdays/ weekend. Other categories contained up to eight subcategories.

Citations were exported into Excel, and evaluated to identify key citations. These key quotes were again analysed in connection with the main topics and form part of this article. Codes comprised the following main themes. Interviews were also analysed according to groups of actors interviewed and according to the protected area type (if relevant).

## Results

### Qualitative Interviews on Conservation Areas and Large Green Spaces

#### Changed use of suburban conservation areas during the COVID-19 pandemic

From the perspective of all experts, the lockdowns resulted in significantly higher numbers of visitors to both conservation areas and urban natural spaces, as the following quote illustrates:

“People’s need for local recreation and green spaces has certainly risen.” (IP3)

In particular, the number of people who did not previously go to forests and fields for recreational purposes increased massively, as did the amount of time they spent in these areas. Those who partake in sports activities, particularly hikers and bikers (mountain bikes and e-bikes), were increasingly present in these areas.

“[…] that more people use natural spaces; whether directly in the forest, in open landscapes, or on agricultural land, it is possible to observe a higher number of visitors. Also, new people or a new group of people can be found outdoors in these places, who did not use to go to a forest for recreational purposes.” (IP1)

As the number of visitors was not counted in several areas, this information was supplied primarily based on direct observation during inspections, forecasts, and estimates, whereby the increase in percentage in comparison to pre-pandemic times was between at least 10% (Purkersdorf Nature Park) and a maximum of 300% (Lainzer Tiergarten biosphere reserve). The following quote shows that this was also significantly different to prior to the pandemic, especially in times of bad weather:

“The number of visitors has increased immensely, I guess three or four times as many, even when the weather is bad.” (IP4)

In some areas, the excessively higher frequency was primarily at weekends and on holidays (e.g., Federal Gardens, Hohe Wand Nature Park), while in other natural spaces there was an equally high number of visitors throughout the whole week during periods of lockdown (e.g., the Vienna Woods biosphere reserve, and sections of the Lainzer Tiergarten). Issues which were especially relevant for conservation areas included the increased number of people observed by the interviewees who chose to get their fill of nature at off-peak times or at night using headlamps in order to avoid the masses (Purkersdorf Nature Park), or temporarily moved their visits to green spaces to other places due to closed food and beverage establishments (Salzwiesen, Maria-Theresia-Platz, Perchtoldsdorfer Heide), which resulted in almost continuous pressure on the surfaces in these locations. Significant seasonal shifts were also noticed, as demonstrated in this quote:

“We also observed that there was now a very high frequency over the winter, which had not been the case before. In the past, before the pandemic, the winter had been a time of respite, during which the vegetation could recover, and since the pandemic, there has hardly been any difference between summer and winter and between the seasons as a whole.” (IP3)

#### Effects of the pandemic on conservation areas

A great number of the challenges faced at the crossroads between conservation and visitor management were further heightened by the pandemic. There was unanimous agreement amongst all experts interviewed regarding the issue of waste, a topic which played a role in all conservation areas and concerned all types of landscapes. In the middle of the forest, in the fields, on open land used for agriculture, in vineyards, and in parks, the amount of waste was enormous during the pandemic. Some wild animals were attracted, which spread food waste and packaging across further patches of land. In addition, hygiene products were deposited in conservation areas, above all masks. The containers people had brought along for their food and drinks were often left behind in grassland areas. This was a predominant occurrence during lockdown phases, as take-away meals were consumed in these areas. There was additional need for staff to collect litter in open spaces, and the lack of waste bins large enough in volume to store the amount of litter thrown away made it necessary to empty them more frequently. Landowners, who are obliged to dispose of waste, sometimes had to remove the larger amounts of litter themselves without additional staff. The following quotes illustrate the pressure on conservation areas and their management:

“… and that then after the picnic the waste, the remnants in the grass, simply stayed there, like pizza boxes, beer cans, etc. Additionally, the challenges for farmers: trampled fields and some kinds of sticks hammered into the ground that stay in there and cause damage to mowers when the grass is being cut…” (IP1)

“There was three times as much, which pushed our resources to their limits. We didn’t get more staff from one day to the next… and our infrastructure isn’t set up for such situations, i.e., number of waste bins.” (IP4)

“The waste situation is a huge topic. Of course, it has always been an issue, but I think it has become heightened due to the pandemic.” (IP6)

“This puts particular strain on the vineyards. The waste is accumulated on private land. The individual landowners are practically obliged to clear up the waste generated by recreation seekers. Farmers report that they first walk around the fields removing the waste before they can mow the grass.” (IP5)

A further problem, which increasingly came to light during the pandemic, is vandalism. Infrastructure for visitors as well as for the purpose of environmental education was damaged. The following quotes illustrate the range of damage done:

“And then it happens that some benches are bashed up, which is more of an issue for the community or for the farmyard, which obviously also results in significant costs, also damage to nature trail signs, etc.” (IP7)

“Vandalism was certainly rife. Yes, a lot is destroyed. In our case, all of the signs were pulled off 13 rod installations. For example, for our forestry use, we have now put banners up that are as wide as the street: Danger due to forest work! Attention, danger to life! Which also get cut down.” (IP2)

“We have box trees there, and these trees are 70/80 years old, are irreversibly destroyed because if the branches are snapped off, holes are created, which don’t grow back in a short amount of time. It is certain that we will have to completely renovate over the next few years, which involves a large investment.” (IP3)

As a result of the lockdowns and the associated measures, such as the closure of sports facilities amongst others, more and more people visited natural spaces close to home to carry out their sports activities. Short-time work models and remote working enabled many people to access ecosystem services in conservation areas or parks for active recreation, commonly in the form of mountain biking, ski mountaineering, hiking, using e-bikes, playing ball games, or running, as the following quote exemplifies.

“Well, recently we have noticed that more mountain bikers have been out riding in conservation area xx… this is simply an amplified trend at present for them to ride through the conservation area, which wasn’t the case before, for example… At the moment, they stick to the paths which people also walk on. As such, it isn’t that dramatic yet. They haven’t created any new paths yet.” (IP9)

According to statements made by several interviewees, however, this resulted in increased conflict with conservation targets due to overuse. In addition, such conflicts were noticeable between the various groups of recreation seekers. Disabled people, children, and elderly people often found themselves confronted with people doing sports. This had a particularly negative impact if the different groups were moving at entirely different speeds.

In order to limit the risk of infection in crowds, many people tried to avoid each other, which led to people leaving paths and new paths being created. This was true both in conservation areas’ central zones and in urban green spaces, where patches of forest and areas of open land, some of which are intended to be untouched habitats for fauna and flora, were walked or ridden on with sports equipment.

“This also has something to do with the fact that many people rediscovered sports and jogging, etc., and that leads to frequency. And this also means that the actual paths are left, and people search for new paths, which means that we suddenly have forest paths that weren’t there previously.” (IP3)

The following quote reflects the opinion of many interviewees that most visitors tried to stick to the designated paths. However, social distancing measures made this difficult and even the behaviour of a smaller number of recreation seekers could cause negative effects if they, as demonstrated in the second quote, pushed ahead in sensitive areas such as central zones, for example.

“90% of people are well-behaved, but the other 10%, they make things difficult. So, what we have is: this is a conservation area, you must stay on pathways, you may not leave the path. And the more visitors there are, the more things like that happen. And at some point, if the path is so busy, people simply look for space and, whether consciously or subconsciously, they get out of each other’s way and leave the paths.” (IP10)

“Previously, illegal routes were also taken in central zones. Now, this has multiplied, routes are downloaded and then walked without checking whether a conservation area is concerned.” (IP2)

Another issue relevant to conservation were those dogs which caused conflicts with other recreation seekers whilst off the lead, but also invoked conflict with the tasks of the management of conservation areas. One quote provides an exemplary illustration that specific consequences were visible in conservation areas.

“For example, in Mauerwald we have the situation that it borders on the Lainzer Tiergarten, a Natura 2000 site, and there has always been a high frequency of dogs. It’s a popular place for people to walk their dogs, like in Hoerndlwald, but in Hoerndlwald there are hardly any roe deer, for example. In Mauerwald there are and there have been several attacks by dogs.” (IP4)

According to the interviewees’ statements, it was possible to determine consequences resulting from the aforementioned changes, which largely heightened the challenges that had already existed. The higher frequency of visitors and larger total number of people who left the designated paths in conservation areas and parks and accessed recreation areas at night, in some areas with their dogs, had implications for wild animals. This was true for wild mammals, such as wild boar and roe deer, and also had an impact on some of the sensitive birds in the breeding period. The following quotes illustrate this:

“The impact of disturbance was most definitely tremendous. I don’t believe that there was a single area in Vienna where there was complete calm.” (IP5)

“As it became busier, the wild animal population increasingly flocked together in places absolutely nobody visited at all. However, interestingly enough, I have seen more wild boar this year than in many of the prior years added together, but it could be that the population has simply grown. When it comes to birds, I can’t imagine that they are affected by this. I think they are relatively robust and, besides, they are able to fly away.” (IP2)

Overall, however, the experts had diverging opinions concerning the threats to endangered species. In some areas, on the one hand, action was consciously taken which damaged rare organisms and negligence also caused impairments. On the other hand, no direct impact on endangered species could be ascertained in several conservation areas since the lockdown situation.

“Thank God we have proof of black stork in the Lainzer Tiergarten, for example, and this is a highly sensitive species. If people move underneath the breeding tree, where the nests are, this interferes with feeding behaviour, etc. In this case, it might be that they fail to breed at the next opportunity. Or a Ural owl which, thank God, has settled there as a result of a resettlement project. This animal is also very sensitive to disruption.” (IP4)

“You can say no because the periods of time during which the intensity soared were relatively short and we do a good job with visitor management, so you can say that we can actually absorb the impact quite well for our species present, so our management plan can be pursued.” (IP7)

“What might be an issue: due to the fact that people really also traipse around the fields and sit down in them or use them for ball games or other things, it could be that there are shifts in species in one field or another. Otherwise, I don’t believe so for individual species as long as they aren’t massively pulling things out, and I haven’t found that people have developed a huge passion for collecting.” (IP2)

The extent to which the intensified visitor streams had an impact on the protection of species was clearly also highly dependent on the individual species and the potential habituation effects as well as the area’s alternatives. During certain seasons and in various localities, due to the spatial and temporal overuse of conservation areas, some organisms did not have access to sufficient undisturbed habitats and opportunities to breed or eat in a natural manner.

“As I said, a kingfisher is relatively tolerant, perhaps it became this way. It simply has to accept that there are so many people around and they accept this, too. Of course they flee, so if we are around, it wouldn’t just perch four metres away, but theoretically it could sit 30 or 40 metres away and this wouldn’t bother it. There can be intermittent overuse. It could be that certain places, where certain species would like to reproduce, are used too heavily and they are then frightened off, or it could be that that collateral damage, so to speak, is caused because orchids are increasingly photographed […] I would say it’s critical between mid-March and mid-May because plants in the meadows here or even insects, they aren’t bothered whether there are more people around right now or not. It’s only critical for birds and perhaps also for a couple of types of mammals. And one bird, for example, we have had it several times in April or at the beginning of May, is the so-called sandpiper and I am convinced that if there were no disruptions, one or the other would have tried to breed and due to the disruptions, it isn’t possible, so to say.” (IP9)

#### Behavioural changes in conservation areas and changed perception of conservation targets during the pandemic

Following critical questioning, most of the experts agreed that there was only limited evidence of greater consideration and increased awareness of nature amongst recreation seekers since the outbreak of the pandemic. As a result of changed time resources, other activities could be carried out which, according to the opinion of individual experts, also led to a changed perception of the areas and their natural resources, as the following quote demonstrates:

“I believe so, because there is a different time budget and there are no alternatives and now I can only focus on a few things and I dedicate myself to them more intensively. This was certainly the case. You only have to look to the forests to see how many tepees have been made out of deadwood and how many children are playing in streams. They haven’t only been using the playgrounds, but they have really made use of the natural environment.” (IP4)

However, the interviewees had differentiated views on whether the influence of spending more time in conservation areas and large green spaces changed people’s perception and appreciation of conservation targets.

“I would say a certain percentage, yes, but I wouldn’t estimate it to be higher than 10%, who might already have been naturally predisposed and have now become a little more attentive. Perhaps this percentage is a little higher.” (IP9)

“I believe that greater attention is paid to nature, but whether this means increased appreciation, I don’t know, well not amongst the wider population, no.” (IP8)

There were visitors who were visibly interested in the natural spaces, who became interested in birdsong, for example, but the functional use of green spaces was at the foreground for many. Critically speaking, conservation areas and large green spaces were primarily viewed as compensation for other places by the experts and served to maximise one’s own personal use or were even treated like a “consumer good”. The following quotes illustrate the spectrum of estimates:

“Of course I notice, people are walking around in a completely different manner. Not everyone, but a noticeable number, I have to say, who more consciously take note of the natural environment, who look at flowers, watch birds, listen to noises, and spend their time in the fields differently.” (IP4)

“I have perceived this only insofar as people are cherishing it more for themselves. However, I don’t have the impression that this is expressed in the form of increased respect for these natural, cultural landscapes, etc.” (IP8)

“That you say, yes, we use it, and it is great to consume it because you see a place as being the place you can relax in. I doubt whether many or most people really consciously appreciate it because it is a valuable ecosystem or whether they see the details as to why it is so worthy of protection and why it is so valuable.” (IP1)

“Now, I don’t see that, that this is common property and belongs to me as well and I can use it as I see fit. People’s perception of nature itself and I want to go for a walk, that is something that has grown. I appreciate this thing and I want to have it and these hiking trips, but the afterthought, that this is also something that needs to be protected or I should take care of it? No! Not to an extent that I could perceptibly verify.” (IP8)

Some interviewees also reported that they assumed that, independent of age, more people had become interested in topics like climate change and environmental issues because media coverage had grown in these areas, as the following quotes indicate:

“That’s difficult to say, but I do think that this whole environment topic is simply really very current and due to this whole climate change, etc., it’s not going to just fade away tomorrow. It’s going to become even more urgent, also in political terms, I think, there is more pressure. Also from the people. And I also think that young people are more vehement than our generation.” (IP6)

The experts were in doubt as to whether the greater amount of time spent outdoors could lead to actual behavioural changes, although it was possible to detect differences between the answers provided by those responsible for the administration of Vienna’s large green spaces and those who manage conservation areas.

“In many cases, I think it was quite simply just going out, being outside, getting out, and this it “mine”, this is the forest I go to now, but creating awareness for the fact that the forest is much more than a recreational area for me—I’m sceptical as to whether this has taken place amongst the population.” (IP7)

“I don’t believe so in this respect, as the types of use are not associated with people being enthusiastic about the surrounding countryside. A large number of these are dog owners, for example. They want to walk their dogs and it doesn’t matter what it looks like or which beauties they are surrounded by. Or the mountain bikers, who don’t look left and right anyway. The runners, who don’t have the time to look left and right either. It’s just about running or being in a natural environment, but not about how it looks.” (IP5)

#### Changed interest in environmental education programmes and conservation activities: outlook on future demand

The answers regarding people’s need to acquire knowledge concerning conservation and related topics do not paint a consistent picture. There were all kinds of responses, ranging from little demand to unchanged commitment right through to a significantly increased desire to find out more about biodiversity loss, ecological assets, and habitats.

“We would need to wait another couple of months to see if this results in changed behaviour outdoors, also in order to get a little feedback from excursion guides as to whether many of the old faces turned out for excursions again as quickly as possible, or whether new recreation seekers in our forests and natural spaces are simply showing more interest after all.” (IP1)

“We are hosting guided herb hikes for the first time this year, this coming weekend, and it was booked up immediately and, funnily enough, by different age groups. This shows that people are taking more of an interest in nature and what it offers, etc.” (IP6)

“Yes, I think we had tours again from May onwards. But there is always a lot of interest, so I haven’t heard that it has increased measurably.” (IP10)

In any case, however, in the time during which there were increased and longer stays in conservation areas, heightened interest in permanent infrastructure for environmental education was observed, as the following two quotes illustrate:

“I do think that people are more attentive to the signs which are located at the sides of the paths.” (IP8)

“The nature trail experienced completely different patterns of use. I hadn’t noticed people reading the display boards in as much detail before as they do now. People have taken more of an interest in those places where they have the right to free use of the forest, like Maurerwald or Schwarzenbergpark.” (IP4)

There are varied assessments as to people’s willingness to volunteer, both in terms of those volunteers who have become newly engaged over the past two years as well as those people who have been committed to conservation for a longer period of time.

“I think things like volunteering days or where people get together to clear a field or things like that are more successful.” (IP2)

“Principally, I would say no because this voluntary work is something which many areas couldn’t survive without if there were no volunteers. This means that we have had those who are really committed involved in various activities for a long time and that’s why it’s a small, exclusive group.” (IP5)

In order to be able to make use of changed appreciation of green spaces as well as increased attention to conservation topics in future, the efforts made by the managers of conservation areas, NGOs and administration institutions will not suffice alone, according to the opinion of several interviewees. In their point of view, not least as a result of the events surrounding the COVID-19 pandemic, political decision-makers from the local level right up to the federal government must become increasingly aware of current and future challenges, e.g., due to climate change, and implement concepts for the strategic development of green spaces, particularly for conservation areas and the management thereof.

“I think that people and society will revert to what they had before relatively quickly. Of course, this is also a question of steering. Political decision-makers are also needed so that this now moves in a different direction. This cannot be made the responsibility of society alone, even though it is very important for people to take care of what’s on their own doorsteps, to make the most of it.” (IP7)

“Fundamentally, I don’t believe this is purely down to the recreation seeker, but politically something has changed and by means of politics, further steps can be defined for recreation seekers. If more attention is given to this topic in the individual districts again because those people living in the districts don’t know what they are causing. Everyone says go out and it is great that so many people are visiting our green spaces, but they don’t know the consequences because they are not directly involved.” (IP5)

“That’s difficult to say, but I do think that this whole environment topic is simply really very current and due to this whole climate change, etc., it’s not going to just fade away tomorrow. It’s going to become even more urgent, also in political terms, I think, there is more pressure. Also from the people. And I also think that young people are more vehement than our generation.” (IP6)

“I think the chances are that people have realised that it is important. I do think so. Particularly when it comes to decision makers, i.e., those responsible for community policy. I do think that it has now become clear how important it is to have something close by and not far away.” (IP9)

The following section complements the perception of the protected areas looking at the Viennese residents and their actual visiting behaviour of green areas as well as their attitude towards them as resource for multiple activities. While the protected area managers were more hesitant regarding a changed perception of the green areas’ contribution to quality of life and their value the quantitative survey presents a clearer picture towards a stronger appreciation of both near-home green areas and large-scale suburban green areas.

### Quantitative Survey Conducted Among the Inhabitants of the City of Vienna and Its Surrounding Municipalities

**Fig. 2:**
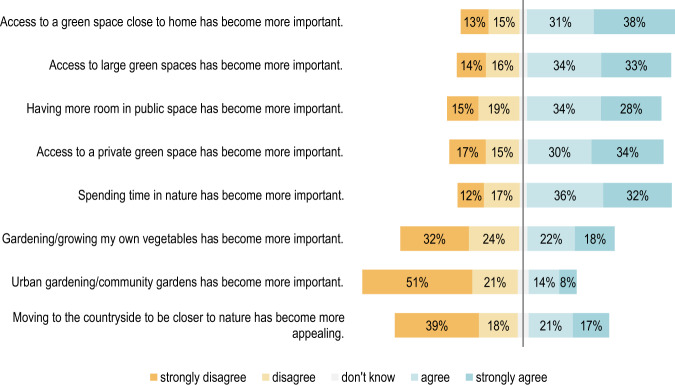
Overview of respondents’ opinions regarding green spaces during the COVID-19 pandemic (*n* = 1012)

The increased meaning of green spaces was not only shown in the interviews, but also in the quantitative survey carried out amongst the population of Vienna and the surrounding communities. Besides the changed use of various types of green spaces, a comparison of the activities before and after the pandemic, and further questions on use of green spaces, the survey also included questions on the perception and importance of green spaces as well as their proximity and accessibility. Figure 2 shows an overview of the statement questions.

On the whole, the results showed significant agreement on the increased importance of spending time in nature or in green spaces. Almost seventy per cent (68%) of respondents either agreed or strongly agreed with the statement *“Spending time in nature has become more important”*. The increased importance of private green spaces due to people’s experience of the pandemic is also highlighted in the results of the survey. More than half of the respondents (64%) agreed or strongly agreed with the statement *“Access to a private green space has become more important”*. Moreover, green and open spaces close to home have also become more important to most of the respondents. Around seventy per cent (69%) indicated that they agreed or strongly agreed with the statement *“Access to a green space close to home has become more important”*. The statement *“Access to large green spaces has become more important”* was agreed with by 34% of the respondents, and 33% strongly agreed. This consensus also illustrates the importance of urban and suburban conservation areas, which are usually more expansive than other types of green spaces in urban areas.

Considering a potential urban exodus trend during the pandemic, the results of the survey showed a comparatively lower level of agreement with the statement: *“Moving to the countryside to be closer to nature has become more appealing”*. In spite of this, around forty (38%) per cent of the study’s participants noted that they either strongly agreed or agreed with this statement.

#### Comparison according to gender

**Fig. 3:**
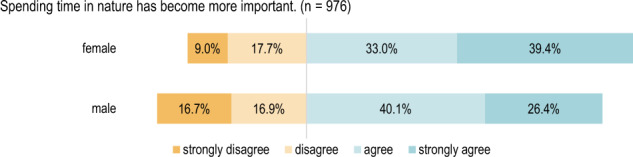
Respondents’ opinions regarding the importance of spending time in nature grouped by gender: Χ²(3) = 26.63, *p* = 7.038e-06, *n* = 976

**Fig. 4:**
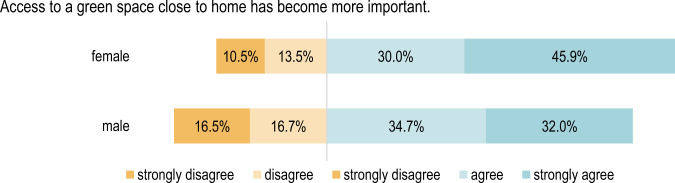
Respondents’ opinions regarding the importance of having access to a green space close to home grouped by gender: Χ²(3) = 21.921, *p* = 0. 00007, *n* = 975

When considering the individual statements according to gender, there is a significant difference (*p* < 0.01) regarding whether it has become more important for the respondents to spend time in natural environments as well as whether it is more important for them to have green spaces close to home (see Figs. 3, 4). Female respondents answered more frequently with “strongly agree”.

**Fig. 5:**
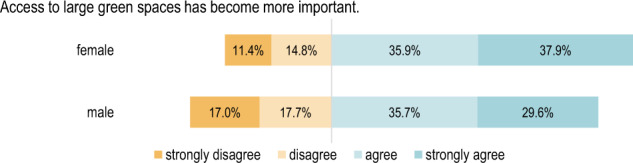
Respondents’ opinions regarding access to a large green space grouped by gender: Χ²(3) = 11.719, *p* = 0.008, *n* = 971

Additionally, concerning the valence of large green spaces, there is a significant difference between men and women (*p* < 0.01) when it comes to their level of agreement with the statement *“Access to large green spaces has become more important”*, as Fig. 5 shows. As a result of their experience of the pandemic, it seems that the importance of large green spaces has changed more distinctively for women than it has for men.

There are also significant differences (*p* < 0.01) between the genders when it comes to the importance of green spaces close to home. Around 46% of female respondents strongly agreed with the statement *“Access to a green space close to home has become more important”*.

#### Comparison by age group

**Fig. 6:**
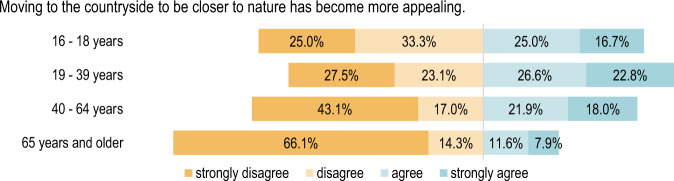
Respondents’ agreement on the increased importance of moving to the countryside grouped by age: Fisher’s exact test, *p* = 1e-07 (2-sided hypothesis), *n* = 960

When evaluating according to age group, it becomes apparent that age has a significant influence on the responses given in connection with several statements. The age groups displayed particularly divergent needs on the topic of moving to more rural areas. Depending on their current phase of life, this does not seem to be important for people aged 65 and over, while the group between 19 and 39 years of age demonstrated much clearer agreement. Almost half of the participants responded to this statement with “strongly agree” or “agree” (see Fig. 6).

**Fig. 7:**
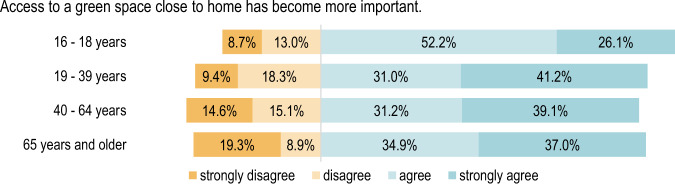
Respondents’ opinions regarding access to a green space close to home grouped by age: Fisher’s exact test, *p* = 0.006 (2-sided hypothesis), *n* = 977

**Fig. 8:**
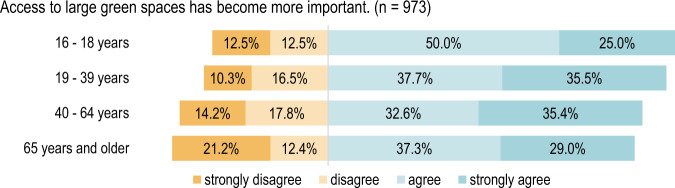
Respondents’ opinions regarding access to a large green space grouped by age: Fisher’s exact test, *p* = 0.031 (2-sided hypothesis), *n* = 973

In addition, the responses to the statements “*Access to a green space close to home has become more important”* (*p* < 0.01) and *“Access to large green spaces has become more important”* (*p* < 0.05) are significantly different according to age group. There is particular divergence between the under eighteens and the remaining age groups. The youngest respondents rated this statement less frequently with “strongly agree”, thus displaying less agreement with it, although almost half of those belonging to this age group gave the answer “agree” (Figs. 7, 8).

People aged above 65 attach less importance to the opportunity to be able to use large green spaces and rate the statement as less applicable than younger respondents aged between 16 and 39.

**Fig. 9:**
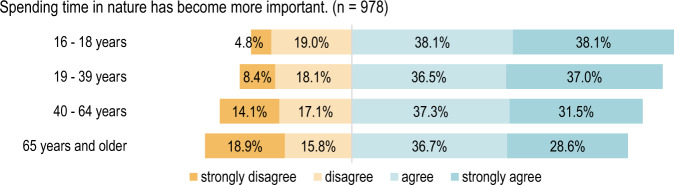
Respondents’ opinions regarding time spent in nature grouped by age: Fischer Fisher’s exact test, *p* = 0.0491 (2-sided hypothesis), *n* = 978

In contrast, there is homogeneous agreement across almost all age groups concerning the statement *“Spending time in nature has become more important”*, as shown in Fig. 9.

#### Comparison by place of residence: Vienna and its surrounding area

**Fig. 10:**
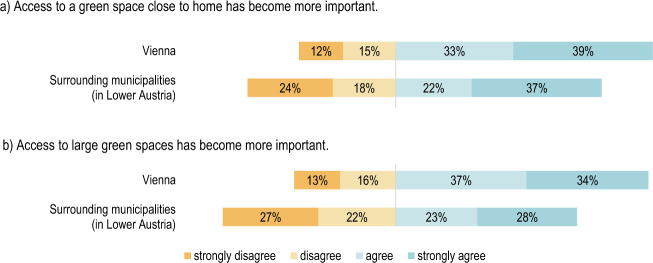
**a** Respondents’ agreement on the increased importance of having access to a green space close to home grouped by state Χ²(3) = 10.919, *p* = 0.012, *n* = 977; (**b**) Respondents’ agreement on the increased importance of having access to a large green space Χ²(3) = 16.300, *p* = 0.001, *n* = 937

There were significant differences between the respondents from the city of Vienna and the communities surrounding Vienna on the topic of whether their experience of the pandemic and the associated measures had changed the importance of large green spaces and of green spaces close to home. More than two-thirds of those in Vienna (71%) awarded the statement “*Access to large green spaces has become more important”* with “agree” or “strongly agree”. In contrast, only slightly more than half (51%) of those from Vienna’s suburbs agreed with this statement. This distribution is in line with the statement regarding the increasing relevance of green spaces close to home, as can be seen in Fig. 10. However, for all other statements, no significant differences could be identified according to place of residence.

**Fig. 11:**
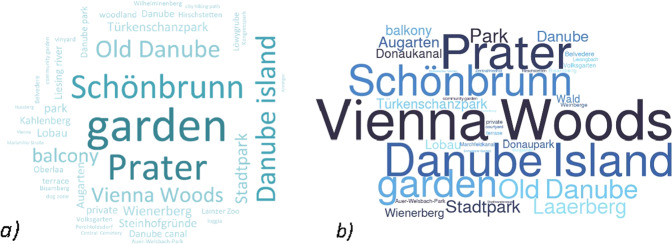
Word cloud containing the names of most visited places during the pandemic, reported by the respondents (*n* = 923) (**a**) Summary of top-1 named destinations (mentioned max. 76 times, min. 4 times); (**b**) Summary of top-3 named destinations (mentioned max. 360 times, min. 10 times)

**Fig. 12:**
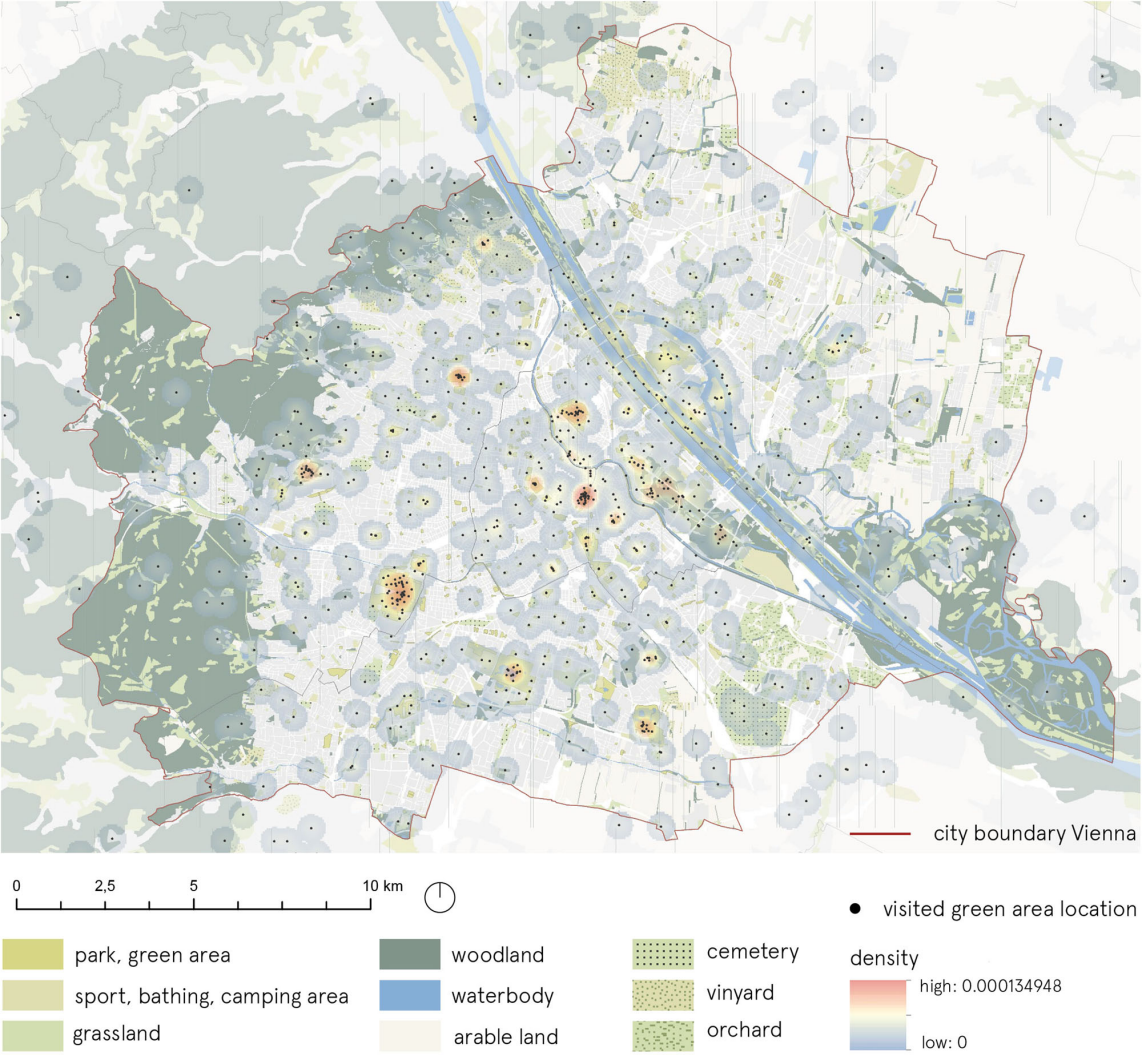
Density map of the most frequently visited places during the pandemic – one most visited place (top-1) reported by the respondents [Geodata credit: basemap.at; Corine Land Cover (CLC) 2018 version 2020_20u1—European Environment Agency (EEA) under the framework of the Copernicus programme—CLC dataset produced with funding by the European Union; city boundary -Stadt Wien – https://data.wien.gv.at; map created using ESRI ArcGIS]

When it comes to preference concerning the most visited green and open spaces during the pandemic (considering one most important destination reported by a respondent; see question 6 in supplementary material S1), 15% of the 923 people answered with a private open space, such as a garden, balcony, or terrace (Fig. 11a). The distribution on the map (Fig. 12), shows that larger, historic parks, such as the grounds at Schoenbrunn Palace, the Augarten, and the Tuerkenschanzpark were often selected as one of the open spaces visited amongst the urban green spaces. In the city centre, it seems that there was a particularly high density of visitors in the Stadtpark and the Volksgarten. Open spaces by the waterside, particularly by the Danube, which plays an important role in the cityscape of Vienna, were also popular. These include open spaces by the Danube Canal, on the Danube Island, and by the Old Danube. Additionally, areas on the banks of the Liesingbach Brook and the recreational area at the Wienerberg with its artificially constructed pond were named as places visited. The density analysis reveals that the concentration of visitors in expansive recreational areas, such as the Vienna Woods or the Lobau, was not quite as pronounced as in the larger parks in urban areas. These areas’ expansiveness enables more even distribution of visitors. Despite the high number of visitors, the structures of the Viennese Prater’s large green landscapes also seem to contribute to a more balanced density distribution.

Considering three most important destinations reported by the respondents, we can see that Vienna Woods were the most popular green area. 39% of the respondents named Vienna Woods among top-3 most visited green areas during the pandemic. Prater, Danube Island and Schönbrunn were mentioned by approx. 15% of the respondents each (Fig. 11b). As the map question was related to the first named destination only, we cannot comment on the exact spatial distribution within green areas related to the second and third mentioned green area.

## Discussion

Overall, the survey confirms challenges by the pandemic for the management and preservation of protected areas but at the same time outlines their high relevance in particular in metropolitan areas. Sustainable development at a local level, maintaining biodiversity, and securing ecosystem services generally require special attention to visitor management in the management of conservation areas (Mandić 2021). In this regard, new challenges emerged due to the somewhat altered visitor flows, both in terms of location and time, during the pandemic. Much of the time ordinarily spent on tours, monitoring activities, and field work had to be invested in hygiene concepts, safety programmes, and preventative measures. These tasks often had to be completed with a highly reduced workforce, as illness and self-isolation as well as cost-cutting measures imposed further negative consequences on conservation (Miller-Rushing et al. 2021). The interviews similarly highlighted the diverse challenges resulting from temporal and spatial changes to visitor flows, litter, vandalism, use beyond the paths, and changed activities for the Austrian conservation areas under investigation. It was revealed that the areas were used differently due to the pandemic and changed time resources. Individual experts pointed out a new approach to nature resulting from new activities. Playground activities were partly moved to other natural environments and larger open spaces were used for longer periods of time. As a result of this, as well as owing to the challenge of trying to adhere to social distancing measures in spite of large number of visitors, it became apparent that visitors also frequently spent time off the network of paths. Sports activities were stepped up and there were also unpleasant encounters between the different groups of users. Individual interviewees also took note of some changed user groups.

As a supplement to studies carried out prior to the pandemic (Fischer et al. 2018), this study confirms that the physical and psychological aspects of socio-cultural activities in urban and peri-urban green spaces continued to be paramount, and the value of the ecological good ‘nature’ and the protected habitats and species per se continued to play a less important role. Due to people’s limited range of movement at times and the closure of indoor facilities, leisure activities were moved to outdoor spaces. In these cases, the conservation areas served as a locality and backdrop, although the experience of nature might have proven to be a positive reinforcer for individual activities, such as yoga. However, several of the interviewees also perceived that there was changed interest in observing nature (e.g., bird watching), even if this only concerned a smaller number of visitors according to their estimates. Moreover, the interviewees confirmed that they were able to observe greater interest in environmental education offers in some areas. The experts attributed more extensive preoccupation with nature trails and other nature education offers to changed time resources. Based upon these interviews, only a limited assessment can be made regarding the extent to which increased visits to conservation areas had an impact on people’s willingness to support activities relevant to nature conservation. In the conservation areas examined, the management had heterogeneous opinions as to whether there could also be changed interest in participating in conservation activities in the future based upon the experiences people had during the pandemic. Internationally, voluntary activities decreased due to the measures taken in the course of the pandemic (Crimmins et al. 2021). Although they could be carried out online to a certain extent, they did not enable the same level of efficient participation as they had in the previous years. Several urban Citizen Science projects, particularly well-established and advertised monitoring programmes, were the only ones able to celebrate strong growth, as shown in the results of a study conducted in the United States (Crimmins et al. 2021). There were diverging assessments on the levels of willingness to commit oneself to voluntary work in Austria.

While the quantitative survey cannot contribute further proof of changed interest in conservation objectives and activities aimed towards their promotion, it does show that the time people spent in natural environments and their proximity to large green spaces in particular have become more important as a result of the pandemic, which supports the assessment of those responsible for conservation areas and large green spaces’ administrative institutions. Growing importance of large green spaces could be also an effect of newly experienced social distancing habits, affecting human comfort distance. Further possible explanation could be an increased interest in outdoor recreation activities, such as hiking, jogging, cycling, during the COVID-19 pandemic (Venter et al. 2020). Thus, large urban parks along with other large open spaces could gain in importance as sport and physical activity destinations, also after the pandemic (Honey-Rosés et al. 2020).

Generally, the study carried out amongst the population of Vienna and the surrounding communities shows that green spaces close to home have become more important. Provisioning good accessibility to green spaces in all urban neighbourhoods belongs to crucial spatial planning challenges within the context of environmental and social justice, as not all citizens have access to a private garden or a high-quality open space within a reasonable distance. Green spaces located close to home, such as neighbourhood parks or semi-public zones offer space for social interaction and recreation. These activities seem to be especially beneficial to city inhabitants during pandemic period, in particular, to social groups, such as children, seniors or other citizen groups with limited mobility. The tendency of increased use of the nearest green areas (<200 m) were observed in Israel, Italy and Spain during COVID-19 pandemic (Ugolini et al. 2020). Similar changes in green space use were also reported in Helsinki, where significant reduction in distances travelled to green spaces were observed (Korpilo et al. 2021). Long-term visitor monitoring in Polish forests revealed differences in recreational use in the subsequent pandemic periods and also showed that observed trends in various forest areas were not always homogenous (Ciesielski et al. 2022). Further studies also confirm the increased importance of having green spaces close to home as the results of surveys carried out in the Cantons of Geneva and Zurich in Switzerland between 9 and 19 April 2020 has shown: The resident population used the open and green spaces on their doorsteps and close to home more frequently and for longer periods of time than usual (Egeter et al. 2020).

The experiences of the pandemic, the measures taken, as well as the somewhat more difficult or limited access to green spaces changed the perception of nature and green spaces for almost seventy per cent of the respondents. The observed increased importance of nearest neighbourhood, time spent in green areas as well access to large natural areas in comparison to the pre-pandemic period complement the assessments made by studies carried out in Australia (Astell-Burt and Feng 2021) concerning the influence of the pandemic on the use of urban green spaces. They confirm results regarding the influence of the pandemic on the perception of green spaces.

In many respects, significant differences were found in the analysis according to demographic factors. The majority of women displayed clearer agreement with statements concerning the changed importance of green spaces as well as access to large green spaces close to home. Additionally, they demonstrated greater agreement regarding the value of spending time in natural environments and green spaces than men. Gender-specific differences in the perception and use of green spaces were already found before the pandemic (2016), e.g. in a Swedish study that examined the use and perception of urban green spaces and came to the conclusion that women tended to use open spaces more frequently and more actively attributed a higher aesthetic value and reported a higher level of perceived well-being in relation with urban green spaces (Ode Sang et al. 2016). At the same time, according to (Ugolini et al. 2020), in Spain and Italy visits during the restrictions fell, in public green spaces more strongly for women than for men. This was the case before the pandemic and it is still mostly women who, according to the traditional lifestyle, overtake more hours of unpaid work, such as childcare and caring for relatives, than men. This inequality worsened during pandemic times, when school closings led to increased work and time expenditure in the household and in childcare (OECD 2020) and this with phased abandonment of public playgrounds (Voglmayr 2020).

Even prior to the pandemic, studies investigating usage behaviour of urban and suburban green spaces found differences according to the participants’ age groups (Ode Sang et al. 2016). When a closer look is taken at the present study’s age groups, significant differences can also be observed. The survey’s very young respondents in particular, as well as elderly people above the age of 65, provide different ratings to people of middle age. This also corresponds with studies based on times of crisis or challenging periods of time, such as heat waves during which elderly residents of Vienna make less use of green spaces than younger age groups (Juschten et al. 2019). The question of whether the pandemic has led to changes regarding the significance of green spaces close to home might be strongly associated with related factors, such as forthcoming relocation in connection with young peoples’ jobs and education as well as the needs of families with young children. A particularly significant difference was determined between younger and older respondents concerning their changed interest to relocate to more rural areas to have more green spaces close to home—an aspect discussed frequently in the media during the pandemic.

A few significant differences were found between opinions of city and peripheral areas residents with regard to their perception of green areas. Viennese population attributed higher importance to large green spaces in comparison to the respondents living in the surrounding communities. In this respect, the quantitative results supplement the observations made in the qualitative study that Viennese residents increasingly made use of green spaces in the surrounding regions during the pandemic and, as such, potentially (re)discovered the value of these green spaces for themselves. The increase in visitor numbers during the period of pandemic restrictions, especially of new visitor groups, which included young people or families with children, is also confirmed by a study from Bonn, which looked at recreation in a sub-urban forest destination (Derks et al. 2020). For some large green areas, such as the large biosphere reserve and the national park at the city’s border usage pressure and impacts might continue also after the pandemic, due to various new housing projects and due to climate change. Lessons learned from the pandemic might be beneficial for instance in times of heat waves (Jiricka-Pürrer et al. 2021). Second regional difference was related to accessibility of green areas. City inhabitants valued green space located close to home more than people living in the surrounding communities. Vienna’s 2025 urban development plan (STEP 2025 for short) sets out the flagship initiative that all people in Vienna should be able to reach the nearest section of the open space network within 250 m of their place of residence. By opening and installing temporary shared space zones in Vienna, the potentially available space for walking and spending time in could also be considerably increased in densely populated and built-up areas of the city (Schrenk et al. 2020). Yet, more research on the social, economic and political driving forces resulting in green space distribution and quality across various regions and cultures is urgently needed (Lopez et al. 2021; Pipitone and Jović 2021). We admit, that the presented case study of Vienna metropolitan area does not fully explore issues related to urban environmental and social justice. Yet, follow up research is planned to advance knowledge on this critical issue.

## Conclusions and Outlook

Our study shows the changed use of urban and suburban large green spaces and conservation areas: first, from the perspective of those responsible for the areas as well as the users (visitors), and secondly, representative of the population of the metropolitan area of Vienna and its surrounding communities. Both perspectives supplement one another. They highlight the somewhat changed use and people’s perception of (large) green spaces in particular.

While the qualitative study deals with the influence of the pandemic and its measures on people’s perception of conservation areas and large green spaces in a more distinctive manner and mostly questions whether there was a change in perception and behaviour in conservation areas, the quantitative study certainly found that there was changed importance in connection with people’s experience of nature and large green spaces close to home. The issue of whether this changed perception will actually remain the same following the pandemic, and will, for example, have an effect on people’s choices concerning place of residence or their future interest in conservation and environmental observation, could be the subject of further studies.

It would be equally appropriate to examine changes in the use of large green spaces, particularly conservation areas, by means of onsite monitoring. Moreover, potentially changed future usage patterns could be surveyed (temporal, spatial, as well as in terms of activities) to establish their influence on these areas, particularly strictly protected areas.

## Supplementary Information

Supplementary Materials
