# Supplementary material for: Experience Them, Love Them, Protect Them—Has the COVID-19 Pandemic Changed People’s Perception of Urban and Suburban Green Spaces and Their Conservation Targets?
Source: Environ Manage. 2022 Oct 12;70(6):1004–22. doi: 10.1007/s00267-022-01721-9 (PMC9555701; doi:10.1007/s00267-022-01721-9)
Supplement: Supplementary file 1 — Supplementary Materials [file 267_2022_1721_MOESM1_ESM.docx]

Supplementary Material (S1)

Examples of survey questions and answers scales used in online survey

**Question Answer Code / Answer Label**

| **Introductory questions (used in quote restrictions)** | | |
| --- | --- | --- |
| 1. **In which state (Bundesland) do you live?**  - **Quote restriction: survey will not be continued if answer = “other” or if number of cases in relevant regions is achieved (quotafull status)** | 1 | Vienna |
|  | 2 | Lower Austria |
|  | 0 | other |
| 1. **In which Viennese district do you live?** |  | Single choice out of list of 23 districts |
| - **Quote restriction: survey will not be continued if number of cases in relevant region clusters is achieved (quotafull status)** |  |  |
| 1. **In which municipality do you live?**  - **Quote restriction: survey will not be continued if municipality = “other” or if number of cases in relevant region clusters is achieved (quotafull status)** |  | Single choice out of list of relevant municipalities |
|  |  | other |
| 1. **Please enter your year of birth.**  - **Quote restriction: survey will not be continued if age category is restricted (<16 years) or if the number of cases in specified age categories is achieved (quotafull status)** |  | number |
|  |  |  |
| 1. **What gender do you feel you belong to?**  - **Quote restriction: survey will not be continued if the number of cases in specified categories is achieved (quotafull status)** | 1 | Single-Choice weiblich |
|  | 2 | männlich |
|  | 3 | anderes |
| **This section refers to the period DURING the pandemic** |  |  |
| 1. **Name the THREE green/open spaces you visit the most (during the pandemic).** |  | Text |
| **6B. Mark the 1st location mentioned (Location 1) on the interactive map. Click on the map and move the map pin (you can also use map zoom to find the location easier)**  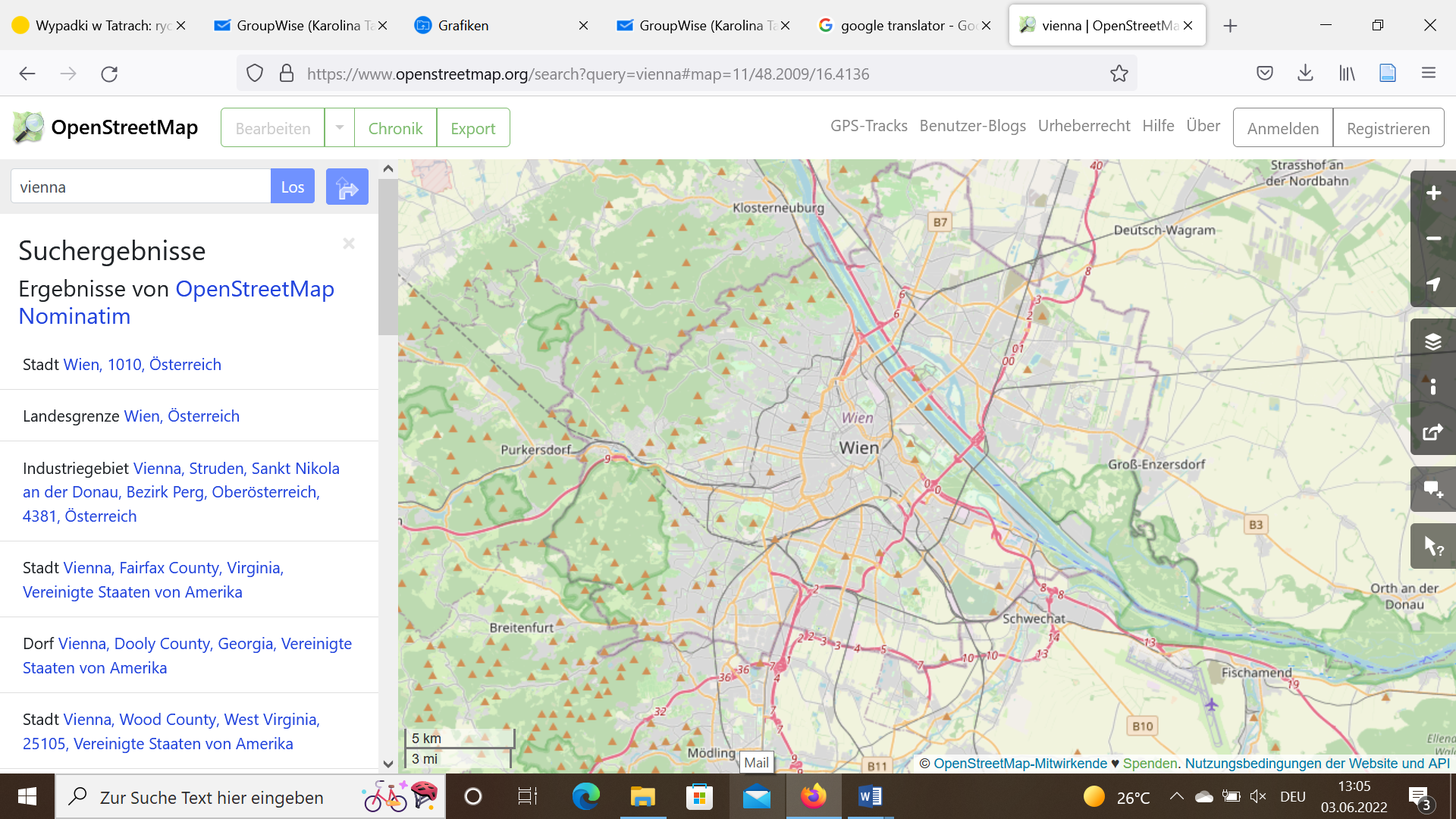 |  | Question with integrated interactive map: Open Street Map |
| 1. **Change in personal attitude/values (List of statements to evaluate)** |  |  |
| 1. **It has become more important to me to have a green space near where I live** 2. **It has become more important to me to be able to visit a spacious green area** 3. **It has become more important to me to have more room in public space** 4. **Having private access to a green space has become more important to me** 5. **Spending time in the countryside and in nature has become more important to me** 6. **Gardening/growing my own vegetables has become more important to me** 7. **Community gardening / urban gardening areas have become more important to me** 8. **The desire to move to a more rural area to have more green space and nature around me has increased** | 1  2  3  4  99 | strongly disagree  disagree  agree  strongly agree  I dont‘ know / I have no opinion |
